# Bioinspired Microcavities Enhancing the Interface of Fe–Carbon Fiber-Reinforced Polymer

**DOI:** 10.3390/ma18235444

**Published:** 2025-12-03

**Authors:** Longfei He, Lianhai Wang, Guorong Cui, Wencong Zhang, Mengkai Chen, Jiabin Hou, Chao Cui

**Affiliations:** 1 School of Shipping, Shandong Jiaotong University, Weihai 264200, China; 2 School of Materials Science and Engineering, Harbin Institute of Technology at Weihai, Weihai 264209, China

**Keywords:** laser micro-drilling treatment, biomimetic, INTERFACIAL properties, molecular dynamics

## Abstract

Laser micro-drilling was applied to Fe substrates to enhance the interfacial properties of carbon fiber-reinforced polymer/iron laminates. This architecture is referred to as a resin-interlocked Fe-CFRP hybrid composite. Inspired by human hair follicles’ exceptional adhesion and filling efficiency, novel biomimetic frustum-integrated cylindrical cavities were engineered for Fe surface modification. Experimental results demonstrate that laser-processed surfaces with varied hole geometries (conical, conical frustum, cylindrical, and frustum-integrated cylindrical cavities) exhibit significantly improved interfacial performance compared to untreated Fe controls. Specifically, RI-Fe/CFRP specimens containing frustum-integrated cylindrical cavities achieved the highest shear strength, with a 44.8% increase over non-drilled counterparts. Subsequent molecular dynamics simulations confirmed the critical role of the cavity geometry, demonstrating that the frustum-integrated cylindrical cavity elevates the Fe–Diglycidyl ether of bisphenol-A interfacial energy and van der Waals interactions by 45.44% and 50.66%, respectively, versus the flat surface. The interfacial energy enhancement mechanism via distinct hole configurations was systematically studied. Furthermore, comprehensive micro-hole topology analysis elucidated the reinforcement mechanism in resin-interlocked Fe-CFRP hybrid composites. Results demonstrate that frustum-integrated cylindrical cavities significantly enhance DGEBA-3,3′-diaminodiphenyl sulfone fluidity during interface simulation, promoting mechanical interlocking and optimized resin-filling efficiency. Laser micro-drilling effectively improves Fe-DGEBA interfacial performance. These findings provide critical insights for designing high-performance composites in aerospace and automotive applications.

## 1. Introduction

Carbon fiber-reinforced polymer (CFRP) [1,2,3] composites offer exceptional advantages, including high specific strength, superior specific stiffness, robust corrosion resistance, and significant design flexibility. They find widespread applications in ships [4], automobiles [5], the aerospace industry [6], and pressure hulls of underwater vehicles [7], as well as in everyday life scenarios. Specifically, in their application in pressure hulls for underwater vehicles, CFRP satisfies requirements for lightweight and high-pressure-resistant materials in deep-sea environments. Researchers are increasingly interested in the application of fiber composite multi-layer structures in the design of shell aircraft cabins [8,9,10] and submarines [11,12]. In recent years, the application of composite materials in the fuselage of Airbus A350 and Boeing 787 aircraft has exceeded 50%. Pressure-resistant shells of various underwater vehicles—including the US deep-sea submersible, China’s Seahawk underwater glider, and Norway’s HUGIN 1000 autonomous underwater vehicle—employ carbon fiber multilayer composites. However, flange connections between shell sections require watertight metal sealing. Safety concerns for underwater carbon fiber structures, underscored by the Titan accident [13], necessitate enhanced interfacial integrity in composite systems. Q235 steel exhibits high strength, low cost, and formability, making it the preferred flange material. Consequently, deep-sea pressure-resistant shells incorporate hybrid CFRP-Q235 steel composite structures.

However, pressure-resistant shells in deep-sea environments endure continuous high pressure, ocean undercurrents, seawater corrosion, and marine biofouling. These conditions induce micrometer-scale interfacial motion between CFRP and Fe, resulting in interfacial loosening and detachment. Microcracks propagate continuously along composite interfaces, significantly degrading waterproof integrity and service life. Regarding CFRP/Fe interfacial issues, Nakazawa et al. [14] investigated the mechanism of adhesion of epoxy resins to various steel surfaces, including cold-rolled steel (CR), galvanized steel (GI), and galvannealed steel (GA). They reported that CR and GA adhesive joints are superior to GI adhesive joints in terms of wet durability and that epoxy resins are chemically adsorbed on zinc oxide and iron oxide through the breaking of the bond between phenoxy oxygen and aliphatic carbon. Xie Guihua et al. [15] studied the effect of adding nano-SiO2-optimized adhesive onto fiber metal laminates (FMLs). Research has found that the addition of nano-SiO_2_ significantly improves the tensile and shear properties of fiber metal laminates, manifesting as higher fracture toughness and higher loading capacity. Li Chuanxi et al. [16] studied the effects of different types of epoxy resin adhesives on the failure mode, adhesion slip relationship, and adhesion strength parameters of CFRP. Meng Cao et al. [17] investigated the effect of graphene nanosheets (GNPs) on the interface and mechanical properties of carbon fiber-reinforced polyether ether ketone-based titanium alloy laminates (CF/PEEK Ti) at the interface between metal and carbon fiber composites. The results showed that the addition of GNPs improved the flexural strength and shear strength of FMLs to some extent. Kai Jin et al. [18] investigated the effect of different laying angles of multi-walled carbon nanotubes (MWCNTs) on the interfacial properties of metal/composite materials using molecular dynamics (MD) simulations. Jiawei Zhang et al. [19] designed a microporous structure on the metal surface to enhance the interface binding force through a mechanical interlocking effect, and at the same time, polydimethylsiloxane improved the interface stress distribution and reduced the interface stripping risk. Hailang Wang et al. [20] prepared a multi-scale micropore morphology on an aluminum alloy surface using a nanosecond pulse laser, studied the influence of geometry size on the wetting behavior of epoxy resin, and successfully applied it to an aluminum alloy-CFRP (carbon fiber-reinforced composite material) heterogeneous anti-collision beam, with bending performance improved by 11%.

Owing to the alternating stacking of Fe and CFRP layers, the interfacial shear strength between metal and resin critically governs the overall performance of RI-Fe/CFRP. To enhance interfacial bonding, research primarily focuses on metal surface treatments that increase roughness, including mechanical processing [21], electrochemical treatment [22], coupling agents [23], and photolithography [24]. However, these methods often lack precise control over the geometry of the surface features, which is crucial for optimizing mechanical interlocking and stress distribution at the interface [25,26]. This represents a significant knowledge gap in the design of metal–composite interfaces. To address this, we introduce a novel laser micro-drilling strategy for Fe surfaces that enables the programmable creation of well-defined micro-cavities. Our work’s novelty lies in the bio-inspired design of specific cavity geometries, particularly the frustum-integrated cylindrical cavity, which is inspired by the exceptional adhesion and filling efficiency of human hair follicles. This approach allows a systematic investigation into how cavity shape governs interfacial performance. Accordingly, RI-Fe/CFRP specimens were prepared by integrating carbon fiber composites with the laser-treated Fe, and their interfacial properties were characterized through shear testing. Furthermore, to unravel the underlying reinforcement mechanisms at the atomic scale, molecular dynamics (MD) [27,28,29,30] simulations were employed to model the interaction between the Fe substrate and the DGEBA [31] epoxy resin within these precisely defined micro-cavities.

## 2. Experiment Details

### 2.1. Materials

Single-lap shear specimens were fabricated using 2 mm thick Fe plates (iron plates (purity: 99.8%) of Grade Q235 were supplied by Taizhou Guixin Metal Products Co., Ltd., Taizhou, China) bonded to a unidirectional carbon fiber/epoxy prepreg (T700-3K, Toray Industries; density: 1.8 g/cm^3^). Diglycidyl ether of bisphenol-A is provided by Weihai Huixing Fiber Products Co., Ltd., Weihai, China. Laser drilling was performed using industrial equipment (supplier: Changzhou Juling Intelligent Technology Co., Ltd., Changzhou, China).

### 2.2. Surface Treatments of Fe Sheets

**Figure 1:**
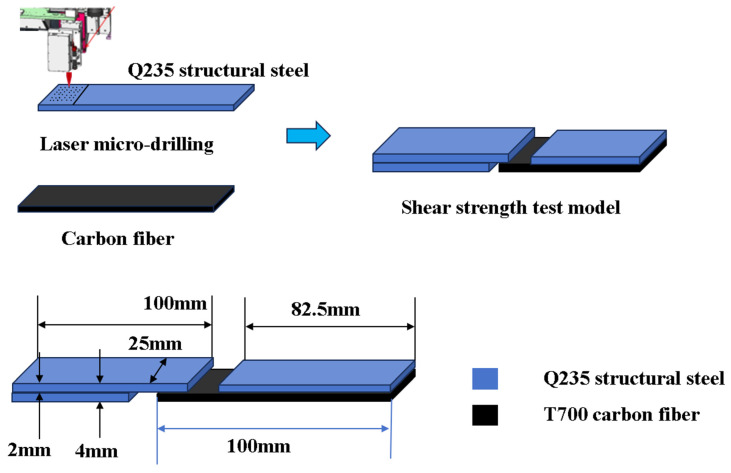
Preparation process and dimensions of shear strength test model.

Fe plate surfaces were mechanically polished, ultrasonically cleaned with anhydrous ethanol, and dried at an ambient temperature. Femtosecond laser processing fabricated geometrically varied micrometer-scale holes within a defined 12.5 mm × 25 mm surface region. Identical hole densities were maintained across all geometric configurations. The resin contact area per drilled hole consistently remains 0.09 mm^2^ across all configurations. For conical holes, the depth measures 0.2 mm with a radius of 0.12 mm. Conical frustum holes exhibit a depth of 0.2 mm, featuring a top radius of 0.09 mm and a bottom radius of 0.05 mm. Cylindrical holes maintain a 0.2 mm depth with a uniform radius of 0.10 mm. Finally, frustum-integrated cylindrical cavities combine a 0.2 mm depth with a top radius of 0.10 mm and a bottom radius of 0.05 mm. The final specimens were rinsed with distilled water and dried at ambient temperature. The production process is shown in Figure 1.

### 2.3. Preparation of Fe-CFRP Hybrid Laminates

The T700 carbon fiber composite layer (thickness: 2 mm; angle: 0°) was laminated onto the Fe plate surface to construct unidirectional RI-Fe/CFRP specimens for single-lap shear testing per ASTM D5868 [32]. Ethanol-treated Fe plates with varied surface modifications were manually layered with carbon fiber prepreg in a sequential stacking sequence. The curing process begins with an increase from room temperature to 120 °C, over the course of 100 min. When the temperature reaches 100 °C during this heating phase, a vacuum is applied. The material is then held at 120 °C under a vacuum for a 120 min curing period. After curing is complete, the system is allowed to cool naturally back to room temperature.

### 2.4. Characterizations

#### 2.4.1. Microscopic Analysis

Fracture surfaces and metal/composite interfaces were characterized using optical microscopy. Quantitative optical microscopy analysis was performed on the damaged interfacial regions of single-lap shear specimens.

#### 2.4.2. Single-Lap Shear Test

**Figure 2:**
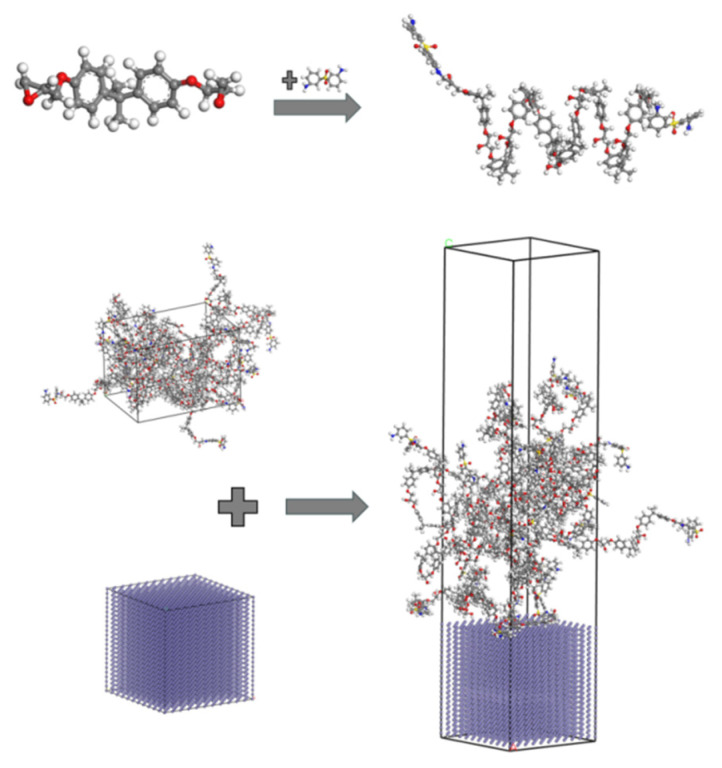
Fe-DGEBA production process.

Shear strength of RI-Fe/CFRP specimens was evaluated per ASTM D5868 [32] at a crosshead speed of 1.5 mm/min. Testing was terminated upon adhesive debonding, with the maximum load recorded. The specimen’s geometry is illustrated in Figure 2, while shear strength (τ_max) was calculated using Equation (1).

(1) $$\tau =F_{m}/(B\times L)$$

Here, τ represents the tensile shear strength, F_m_ represents the maximum test force, B represents the width of the bonding area, and L represents the length of the bonding area. Five distinct laser-treated configurations (conical, conical frustum, cylindrical, frustum-integrated cylindrical cavity, and non-drilled control) were tested. A total of 3 replicates per configuration were tested, yielding a total of 15 experimental measurements to ensure statistical reliability.

## 3. Molecular Dynamics Analysis

### 3.1. Molecular Dynamics Models

**Figure 3:**
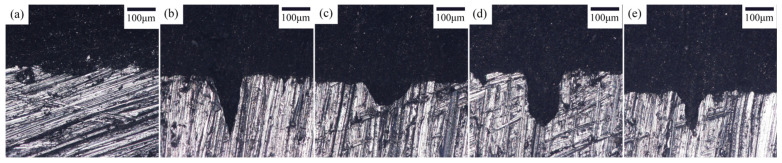
Optical microscopy micrographs of Fe-DGEBA interface: (**a**) prototype; (**b**) conical hole; (**c**) conical frustum-shaped hole; (**d**) cylindrical hole; (**e**) frustum-integrated cylindrical cavity hole.

**Figure 4:**
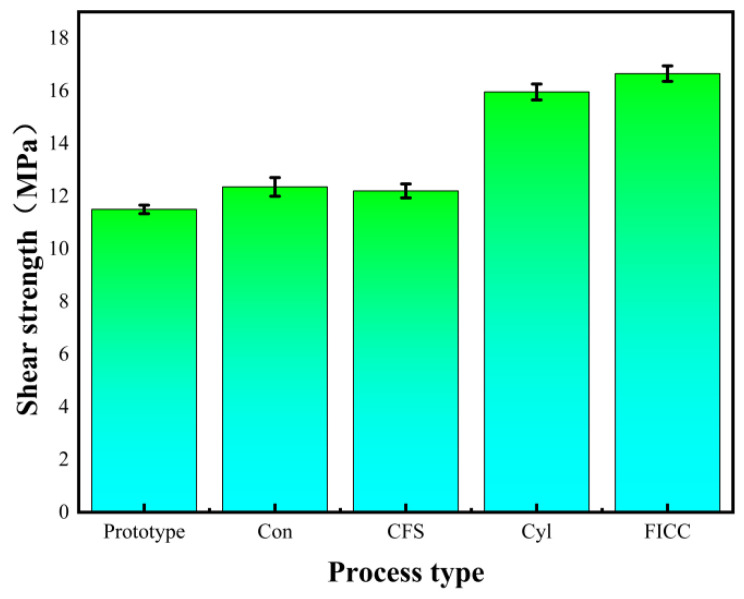
Shear strength test results for RI-Fe/CFRP. Abbreviations: Con (conical), CFS (Conical frustum-shaped), Cyl (cylindrical), FICC (frustum-integrated cylindrical cavity).

**Figure 5:**
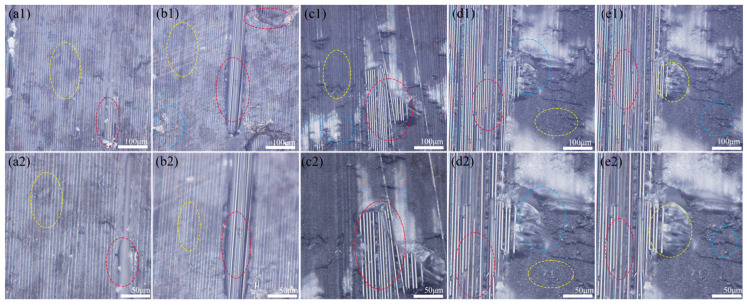
Optical microscopy micrographs of Fe-DGEBA cross section: (**a1**,**a2**) prototype; (**b1**,**b2**) conical hole; (**c1**,**c2**) conical frustum-shaped hole; (**d1**,**d2**) cylindrical hole; (**e1**,**e2**) frustum-integrated cylindrical cavity hole. Instructions: Post-failure analysis of single-lap shear specimens. Failure modes were characterized through surface morphology examination: (**a1**,**a2**) adhesive failure (debonding) at the Fe/resin interface, characterized by a smooth, laser-textured Fe surface with minimal resin residue (outlined in yellow); (**b1**,**b2**) cohesive failure within the CFRP laminate, identified by exposed carbon fibers and resin imprints on the counterpart surface (outlined in red); (**c1**,**c2**) mixed-mode failure, exhibiting concurrent adhesive and cohesive failure regions on a single fracture surface (outlined in blue).

Molecular dynamics simulations were employed to overcome experimental constraints in investigating the effects of surface drilling on the interfacial properties of a Fe–Diglycidyl ether of bisphenol-A (DGEBA). The modeling procedure involved the following: (1) construction of a 3 nm × 3 nm × 13.5 nm computational domain containing Fe and DGEBA, (2) synthesis of DGEBA polymer with 8 repeating units, (3) cross-linking of 3,3′-diaminodiphenyl sulfone (3,3′-DDS) to terminal DGEBA sites forming DGEBA-DDS monomers, (4) generation of a simulation box containing 10 DGEBA-DDS chains (Figure 3), (5) creation of a 3 nm × 3 nm × 3 nm Fe (001) supercell, (6) interface assembly within a 3 nm × 3 nm × 10.5 nm domain with a 3 nm vacuum layer to eliminate surface interactions, and (7) fixation of bottom-layer Fe atoms and insertion of 10 DGEBA-DDS chains. Perforation effects were evaluated using five geometrically modified configurations (Figure 4) and an untreated control. All models employed the COMPASS force field [33] for particle interactions, validated for metal–organic interfaces. The COMPASS force field was utilized, which is well-established for simulating organic and polymeric systems such as the DGEBA epoxy resin and DDS curing agent used in this study, ensuring the accurate predictions of interfacial interactions and mechanical properties.

The interfacial adsorption behavior was analyzed via MD simulation. The simulation protocol comprised the following steps. (1) Energy minimization: This involved an initial relaxation of the Fe-DGEBA system to stabilize potential energy. (2) Curing simulation (NVT ensemble): This involved a temperature ramp: 273 K → 393 K (100 ps, 1 fs timestep), with full-precision trajectory recording. (3) Isothermal equilibration (NVT): A constant temperature was applied, 393 K (100 ps, 1 fs timestep), with full-precision trajectory recording to enhance epoxy-Fe integration. (4) Cooling phase (NVT): The temperature was reduced, 393 K → 273 K (30 ps, 1 fs timestep), with full-precision trajectory recording. The COMPASS force field was implemented throughout all stages.

### 3.2. Equations of Energy

In this study, interfacial energy was analyzed and calculated through MD simulations, with the total potential energy of the system determined using Equation (2).

(2) $$E_{total}=E_{valence}+E_{crossterm}+E_{non-bond}$$

E_total_ is the total potential energy of the system, E_valence_ is the chemical bond interaction energy, E_crossterm_ is the cross-term interaction energy, and E_non−bond_ is the non-chemical bond interaction.

Non-chemical bonding interactions were calculated according to Equation (3).

(3) $$E_{non-bond}=E_{vdw}+E_{coulomb}+E_{H-bond}$$

Here, e_vdw_ is the van der Waals interaction energy, e_coulomb_ is the electrostatic interaction energy, and e_h-bond_ is the hydrogen bonding interaction energy.

Here, the interface energy of the Fe-DGEBA system is calculated as follows:

(4) $$\Delta E_{Fe\&DGEBA}=E_{Fe-DGEBA}-(E_{Fe}+E_{DGEBA})$$

where ΔE_Fe&DGEBA_ denotes the interface energy between the Fe plate and the DGEBA, and E_Fe−DGEBA_, E_Fe_, and E_DGEBA_ are potential energies of Fe-DGEBA, Fe, and DGEBA.

## 4. Results and Discussion

### 4.1. Microstructure Analysis

The laser drilling parameters for Fe surfaces are detailed in Section 2.2, demonstrating suitability for metal surface treatment. Figure 3 presents optical micrographs of interfaces with different laser-perforated geometries at identical magnification. Figure 3a depicts the untreated Fe–epoxy interface, revealing a roughened Fe surface devoid of resin adhesion. Surface cavities indicate interfacial strength defects between Fe and epoxy resin.

Figure 3b–e present optical micrographs of interfaces for conical, conical frustum, cylindrical, and frustum-integrated cylindrical cavity laser-perforated Fe plates, respectively. These micrographs exhibit defect-free interfaces, confirming continuous physical bonding. Surface perforation enhances the Fe–epoxy contact area, improves interfacial mechanical interlocking, and thereby optimizes bonding performance.

### 4.2. Interlaminar Shear Properties of Fe-CFRP Hybrid Laminates

Lap shear testing determined the interfacial adhesion strength of RI-Fe/CFRP to verify micro-drilling effects on interface performance, with results presented in Figure 4. Surface micro-drilling enhanced the shear strength of RI-Fe/CFRP compared to untreated specimens. Different hole geometries influenced shear strength distinctively. Conical drilling treatment yielded 12.5 MPa shear strength, while conical frustum drilling produced 12.6 MPa. Cylindrical drilling treatment achieved 16.2 MPa shear strength. The frustum-integrated cylindrical cavity treatment attained maximum shear strength at 16.8 MPa. Untreated specimens exhibited 11.6 MPa shear strength. Performance enhancement percentages measured 7.8%, 8.6%, 39.7%, and 44.8%, respectively, for these configurations.

Figure 5 presents optical micrographs of Fe surfaces post single-lap shear testing. Figure 5(a1–e2) correspond to untreated control, conical, conical frustum, cylindrical, and frustum-integrated cylindrical cavity treatments, respectively. Figure 5(a1,a2) depicts the untreated Fe surface exhibiting minimal resin adhesion. Figure 5(b1–e2) show conical, conical frustum, cylindrical, and frustum-integrated cylindrical cavity treated surfaces. Notably, in Figure 5(e1,e2), epoxy resin residues (red circles) remain within perforations, demonstrating micro-mechanical interlocking between Fe surface topology and epoxy matrix.

Figure 5 presents optical micrographs of carbon fiber composite surfaces post-single-lap shear testing. Figure 5(a1,a2) depict CFRP surfaces in untreated Fe controls, exhibiting dominant Fe–epoxy interfacial debonding failure (yellow circles) at the metal–adhesive interface, indicating inadequate bonding strength. Figure 5(b1,b2) show conical laser-drilled Fe specimens with two failure modes: Fe–epoxy debonding (yellow circles) and epoxy–fiber matrix failure in perforated regions (red circles), suggesting partial interfacial enhancement. Figure 5(c1,c2) illustrate conical frustum-treated surfaces demonstrating tri-modal failure: Fe–epoxy debonding (yellow), epoxy–fiber failure (red), and minor mixed-mode failure (blue circles), confirming moderate interfacial improvement. Figure 5(d1,d2) reveal cylindrical-drilled specimens dominated by epoxy–fiber failure (red circles) with secondary Fe–epoxy debonding (yellow) and mixed failure (blue), indicating significant bond strength enhancement. Notably, Figure 5(e1,e2) exhibit frustum-integrated cylindrical cavity treated surfaces with predominant epoxy–fiber matrix failure (red circles), demonstrating substantial interfacial reinforcement. This failure mode shift confirms the efficacy of composite drilling treatment in enhancing Fe–epoxy bonding, thereby supporting mechanical performance improvement in metal–CFRP composites. The frustum-integrated cylindrical cavity yielded the highest interfacial shear strength of 16.8 MPa in this work. This finding aligns with the established understanding that mechanical interlocking, achieved through specific surface geometries, is a highly effective mechanism for enhancing metal–polymer interfaces [34]. The consistency between these results underscores the broad applicability of this reinforcement strategy.

The 44.8% improvement in shear strength achieved by our bio-inspired microcavities presents a compelling alternative to nanoparticle-enhanced interfaces. This enhancement is comparable to, and in some cases surpasses, the performance gains reported for composites reinforced with multi-walled carbon nanotubes (MWCNTs). For instance, studies have shown that the interfacial adhesion is maximized when MWCNTs are oriented at specific angles (e.g., 45° or 90°) to the interface, which maximizes the contact area and mechanical interlocking with the polymer matrix [18].

While both strategies—our micro-scale cavitation and nanoscale MWCNT reinforcement—aim to enhance interfacial properties, the underlying mechanisms exhibit distinct advantages. In contrast, our bio-inspired structures operate on a micro-scale, creating a continuous and deterministic mechanical interlock that is less susceptible to issues of dispersion and agglomeration commonly associated with nanofillers. More importantly, as our MD simulations revealed, the optimized cavity geometry not only provides macroscopic anchorage but also fundamentally promotes resin fluidity and maximizes van der Waals interactions at the molecular level. This represents a design-led approach to interface control, offering a potentially more reproducible and scalable pathway for enhancing interfacial strength in structural composites.

### 4.3. Molecular Dynamics Simulation Results

**Figure 6:**
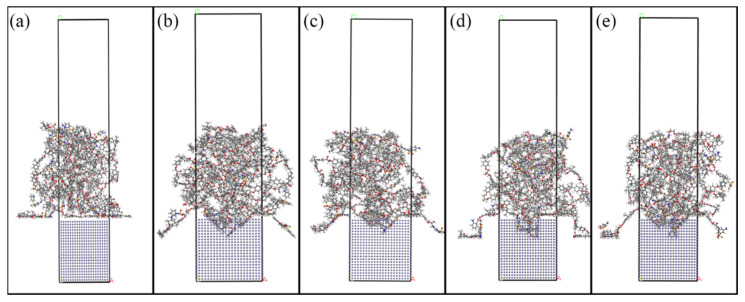
Fe-DGEBA system MD simulation models.: (**a**) prototype; (**b**) conical hole; (**c**) conical frustum-shaped hole; (**d**) cylindrical hole; (**e**) frustum-integrated cylindrical cavity hole.

Molecular dynamics simulations reveal markedly different DGEBA adsorption behaviors contingent on Fe surface topology. Undrilled Fe surfaces exhibit sparse molecular adsorption (Figure 6a), whereas laser-drilled configurations demonstrate dense DGEBA accumulation, evidenced by density distributions in Figure 6b–e.

Molecular dynamics simulations reveal that laser-drilled Fe surfaces significantly enhance interfacial energy compared to untreated controls, calculated via Equation (4) (Figure 6). DGEBA adsorption primarily occurs through van der Waals interactions, driving molecular adhesion to Fe layers. Figure 6 illustrates how different drilling geometries affect interfacial compatibility. Simulations confirm that surface drilling improves Fe–epoxy adhesion via mechanical interlocking, consequently optimizing interface performance. Hole morphology critically influences DGEBA molecular arrangement and contact area. Analysis indicates substantial interfacial enhancement from drilling treatments, with composite–hole configurations outperforming others. Quantitatively, the untreated Fe-DGEBA system exhibits −3653.99 kcal/mol interfacial energy (adsorption direction). Conical drilling increases this to −4130.37 kcal/mol (+476.38 kcal/mol). Conical frustum treatment yields −3973.94 kcal/mol (+319.95 kcal/mol). Cylindrical perforation achieves −5118.34 kcal/mol (+1464.35 kcal/mol). Crucially, frustum-integrated cylindrical cavities maximize improvement at −5274.59 kcal/mol (+1620.60 kcal/mol), demonstrating optimal interfacial reinforcement.

### 4.4. Mechanism of Surface Drilling Treatment on Interface Strengthening

Single-lap shear testing confirmed significant reinforcement effects from Fe surface micro-drilling. Consequently, molecular dynamics simulations were employed to elucidate interfacial strengthening mechanisms. The influence of hole geometry on interface enhancement remains incompletely characterized. To address this knowledge gap, four distinct drilling configurations—conical, conical frustum, cylindrical, and frustum-integrated cylindrical cavity—were simulated.

MD simulations demonstrate that distinct Fe surface drilling geometries significantly alter molecular configurations and interfacial energies within the system. DGEBA molecular distribution and population density on Fe layers vary according to perforation topology. Concurrently, the quantity of adsorbed DGEBA molecules exhibits drilling-type dependence. Maximum adsorption capacity occurs with frustum-integrated cylindrical cavity geometries.

**Figure 7:**
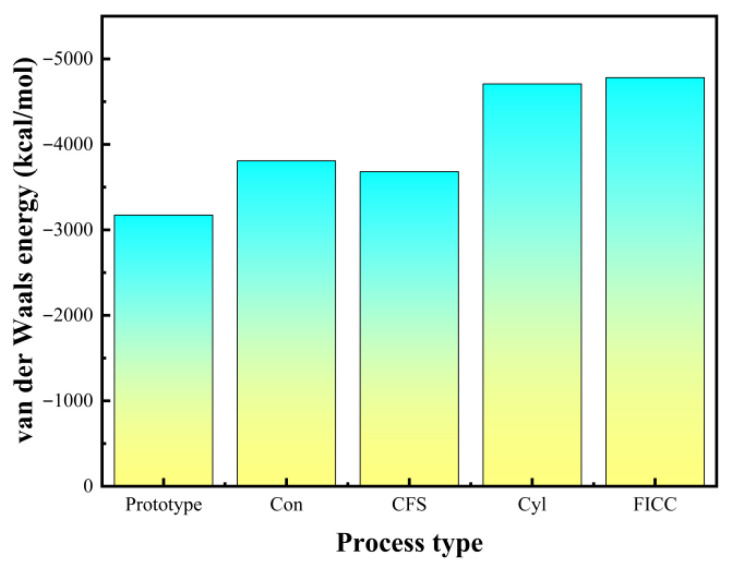
Average van der Waals energy of the last 20 frames at different holes.

Figure 7 demonstrates significantly enhanced van der Waals forces after Fe surface drilling, indicating substantial bond strength improvement. DGEBA polymer chains conformationally adapt to drilled Fe topologies (Figure 6a–e). Interfacial energy analysis confirms van der Waals interactions dominate adsorption mechanisms, with perforation treatments markedly increasing these forces.

**Figure 8:**
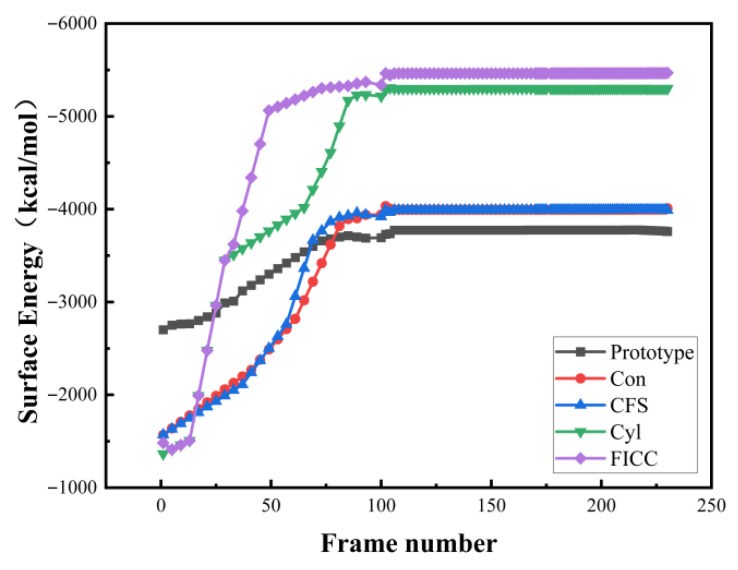
Interfacial energy evolution during curing process.

**Figure 9:**
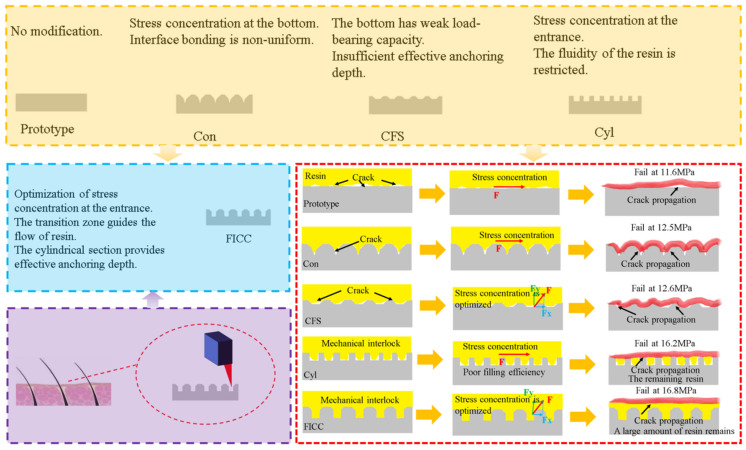
Schematic illustrating the multiscale interfacial enhancement mechanism enabled by bioinspired hair-follicle microcavities.

Molecular dynamics simulations employing total energy analysis (Figure 8) reveal that Fe surface drilling treatments fundamentally alter DGEBA molecular distribution and interfacial configurations. Van der Waals interactions measure −3173.287 kcal/mol for untreated surfaces. This increases to −3807.703 kcal/mol with conical holes, −3680.725 kcal/mol with conical frustum holes, −4708.007 kcal/mol with cylindrical holes, and peaks at −4780.762 kcal/mol for frustum-integrated cylindrical cavities. Drilled surfaces exhibit expanded DGEBA-Fe contact areas compared to untreated systems. Molecular distribution patterns vary significantly across perforation geometries, with frustum–cylindrical composite holes achieving maximal DGEBA adsorption density. Consequently, intermolecular interaction energies and interfacial strengths critically depend on perforation topology. Mechanism of Surface Drilling Treatment on Interface Strengthening (Figure 9).

## 5. Conclusions

This study investigates laser micro-drilling effects on RI-Fe/CFRP interfacial performance for geometrically distinct Fe surfaces (conical, conical frustum, cylindrical, frustum-integrated cylindrical cavity). Molecular dynamics simulations verified interfacial enhancement mechanisms through surface drilling. Single-lap shear testing of Fe-CFRP laminates and MD simulations of Fe-DGEBA interfaces elucidated how micro-perforations improve epoxy mobility, thereby enhancing mechanical interlocking. Thus, the following conclusions are drawn:

(1) Laser micro-drilling on Fe surfaces effectively enhances Fe-DGEBA interfacial and mechanical properties. The frustum-integrated cylindrical cavity yields 44.8% higher shear strength than non-drilled RI-Fe/CFRP.

(2) MD simulations reveal that van der Waals forces primarily govern Fe-DGEBA interfacial performance. With frustum-integrated cylindrical cavities, van der Waals interactions increase by 50.66% compared to untreated Fe surfaces.

(3) MD simulations confirm that Fe surface micro-drilling enhances Fe-DGEBA interfacial energy and RI-Fe/CFRP properties. The frustum-integrated cylindrical cavity increases interfacial energy by 45.44% compared to untreated systems, demonstrating effective interface enhancement.

## Data Availability

The original contributions presented in the study are included in the article. Further inquiries can be directed to the corresponding authors.
